# The Risk Monitoring of Aflatoxins and Ochratoxin A in Critical Control Point of Soy Sauce Aroma-Type Baijiu Production

**DOI:** 10.3390/toxins13120876

**Published:** 2021-12-08

**Authors:** Siyu Zhang, Song Liu, Wenwen Zeng, Weiyun Long, Ye Nie, Yan Xu, Fan Yang, Li Wang

**Affiliations:** 1 Kweichow Moutai Co., Ltd., Renhuai 564500, China; zsy@moutai.com.cn (S.Z.); ls@moutai.com.cn (S.L.); zww@moutai.com.cn (W.Z.); lwyMT@moutai.com.cn (W.L.); ny@moutai.com.cn (Y.N.); 2 State Key Laboratory of Food Science and Technology, Synergetic Innovation Center of Food Safety and Nutrition, The Key Laboratory of Industrial Biotechnology of Ministry of Education, School of Biotechnology, Jiangnan University, Wuxi 214122, China; yxu@jiangnan.edu.cn

**Keywords:** mycotoxins, soy sauce aroma-type baijiu, UPLC-MS/MS, daqu

## Abstract

Soy sauce aroma-type baijiu-producing regions are mostly in southwest China (Guizhou and Sichuan province) with a hot and humid subtropical monsoon climate, which is conducive to the propagation of toxigenic fungi. This suggests that there is a risk of potential contamination by mycotoxins in the soy sauce aroma-type baijiu production process, which poses significant food safety risks. Few studies on the safety of mycotoxins in soy sauce aroma-type baijiu production exist. Aiming to evaluate the safety of mycotoxins in soy sauce aroma-type baijiu during its production, this study screened and analyzed mycotoxic risk at critical points throughout the production process, investigated from raw materials, daqu, alcoholic fermentative grains, crude baijiu and microbial communities in different stages of the production process. The aflatoxins (AFs) and ochratoxin A (OTA) contents in wheat, daqu, alcoholic fermentative grains and crude baijiu samples were detected by ultra-performance liquid chromatography with tandem mass spectrometry. Mycotoxins were detected in wheat, daqu and alcoholic fermentative grains. The AFs and OTA detection rates, as well as their contents in the daqu samples, were relatively higher compared to those observed in the wheat and alcoholic fermentative grains. AFs were detected in 30% of the daqu samples, while OTA was detected in 20% of the daqu samples, though the contents of both AFs and OTA were under the maximum limit of the Chinese national standard. Furthermore, the fungi contained in daqu samples were isolated and identified, and the results showed that no fungi in the separated bacterial strains were producers of mycotoxins. According to the assessment results, the safety of soy sauce aroma-type baijiu production process in terms of AFs and OTA is confirmed.

## 1. Introduction

Mycotoxins are secondary metabolites produced by certain genera of filamentous fungi that grow on cereals and cereal products [1]. Most of the mycotoxins have a carcinogenic, teratogenic, and mutagenic effect, among which aflatoxins and ochratoxin A are the most representative [2,3]. Also, Aflatoxins (AFs) and ochratoxin (OTA) represent the groups of mycotoxins that are predominantly analyzed in cereals and alcoholic beverages [4,5]. Provided that favorable growth conditions exist, toxigenic fungi can produce mycotoxin contamination during production, storage, processing, and distribution of cereals [6,7]. When the raw material is contaminated during the elaboration, mycotoxins can be present in the final products [8,9]. It is necessary to implement risk management and monitoring of mycotoxins in the whole production chain that guarantee the safety of the final products.

Unique in representing the crystallization of traditional Chinese culture, baijiu has a long history that extends backward more than 2000 years. Essentially, there are 12 types of baijiu, including soy sauce aroma-type baijiu, Nongxiangxing baijiu, Qingxiangxing baijiu, Mixiangxing baijiu and so on [10]. Exceptionally pure and mild, soy sauce aroma-type baijiu is a typical representative of Chinese baijiu. The majority of soy sauce aroma-type baijiu distilleries are distributed in southwest China. The hot and humid subtropical monsoon climate of this special region provides sufficient water resource and a perfect environment for the production of soy sauce aroma-type baijiu. However, this environment is also beneficial to fungal multiplication and growth because mycotoxins are one of the foodborne hazards mostly susceptible to climate change [11,12], of which high temperatures is a major factor for OTA contamination [13]. This indicates potential mycotoxins contamination risk in the production-process of soy sauce aroma-type baijiu.

**Figure 1:**
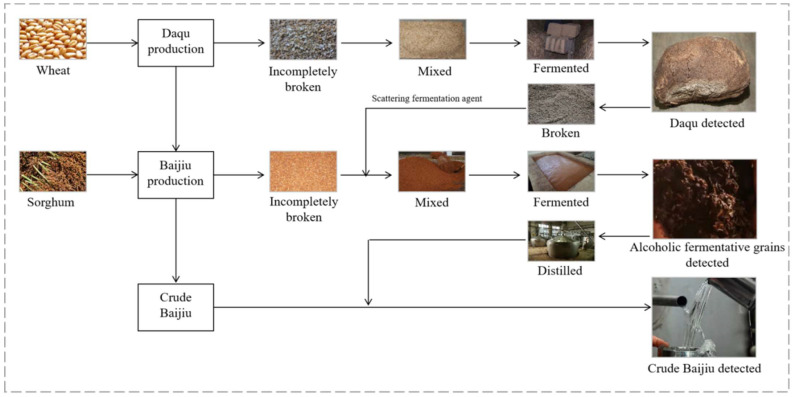
A selection of production techniques for soy sauce aroma-type of baijiu.

The production technology of soy sauce aroma-type baijiu is complicated and contains processes of fermentation, distillation and storage [14,15]. Typically, wheat is broken down—incompletely at first—and mixed with water and a ripe starter for inoculation. The mixed raw materials are made into a square block, which then undergoes stacking and fermentation processed, at a high temperature. The product obtained, after complete fermentation, is called daqu. daqu is rich in glucoamylase, which can decompose starch in the alcoholic fermentative grains into sugar. Sugar is then transformed into alcohol by yeast. Finally, crude baijiu is obtained by distilling alcoholic fermentative grains. Thus, the process of soy sauce aroma-type baijiu production includes the critical steps of wheat, daqu, alcoholic fermentative grains, and crude baijiu (Figure 1). Through the hazard analysis critical control point (HACCP) quality management system [16], the potential mycotoxic hazard risks of the critical control point in baijiu production can be analyzed.

In recent years, many methods have been developed to detect the content of mycotoxins in samples (raw materials, daqu, alcoholic fermentative grains, crude baijiu) of the production process for alcoholic beverages. A variety of methods for detecting OTA and AFs in foods are specified in Chinese national food safety standards. These include isotope dilution liquid chromatography tandem mass spectrometry, immunoaffinity chromatography purification liquid chromatography tandem mass spectrometry and enzyme-linked immunosorbent screening [17,18]. In 2016, a novel quantitative analytical methodology, solid-phase extraction, and high-performance liquid chromatography (SPE-HPLC) were developed and validated to detect OTA in Chinese baijiu by the Xu group. The 76 soy sauce aroma-type baijiu formulations in different geographical regions of China were analyzed by this method, of which OTA was detected in 9 samples, with a maximum concentration of 170 µg/L [19]. In 2017, Xu et al. adopted solid-phase extraction (SPE) combined with ultra-performance liquid chromatography (UPLC-MS/MS) to analyze the OTA content of 133 daqu samples, which were taken from different regions of China. The results showed that about 50% of the samples were contaminated by OTA [20]. In 2017, the level of AFs, AFB1 and OTA in wheat flour produced in a province of Kurdistan was measured by enzyme-linked immunosorbent assay (ELISA) by Keiwan et al.; the level of AFs, AFB1 and OTA exceeded the maximum limit established by the EU in 68.18, 90.91 and 36.36% of the samples, respectively [21]. The content of ochratoxin A in wheat samples from various Algerian regions was detected by a high-performance liquid chromatography-fluorescence detector (HPLC-FLD), as reported by Saliha et al. in 2018. The results showed that 77% of wheat samples were contaminated by ochratoxin A, with a pollution amount of 0.84~34.75 µg/kg [22]. The QuEChERS (quick, easy, cheap, effective, rugged and safe) method is effective for the analysis in a wide variety of complex matrices [23]. In 2018, the Zhao group used an improved QuEChERS method to treat 32 wheat samples purchased from the supermarket and conducted quantitative analysis by liquid chromatography-tandem mass spectrometry (LC-MS/MS). It was found that six wheat samples contained AFB1, with an average content of 0.06 µg/kg [24]. However, risk screening and assessment for mycotoxins in critical points of the soy sauce aroma-type baijiu production is not usually considered in the literature. In addition, the safety of baijiu, in terms of mycotoxins, over its whole production has not been studied.

In this study, a complete set of food safety evaluation systems for baijiu production is developed. Here, AFs and OTA concentrations in a range of intermediate and final products, ranging from raw materials to base baijiu, and covering critical points in the production process were analyzed. The safety associated with microorganisms identified in positive samples was further evaluated through microbial isolation and identification.

## 2. Results and Discussion

### 2.1. AFs and OTA Content in Wheat Samples

Wheat is a major raw material for daqu production; AFs and OTA contamination may occur during storage, due to improper post-harvest handling [25]. So it is necessary to monitor the content of mycotoxins in wheat at its origin. AFs (B1, B2, G1, G2) and OTA content of 10 wheat samples from different storage facilities, used in production, were examined by the UPLC-MS/MS method (Table 1). The results are listed in Table 1. Ten of the tested wheat samples were not contaminated with AFs. On the other hand, only one wheat sample was positive for OTA (1.94 μg/kg) out of the ten tested, the content of which was far lower than the maximum limit standards. In 2006, the maximum limit of OTA in grains was 5 μg/kg, as established by the Commission of the European Communities [26]. Thereafter, the same maximum limit was specified by the Chinese national standard in 2017 [27].

In 2014, Li et al. carried out surveys and monitoring projects to determine the occurrence of mycotoxins in agro-products in the Yangtze Delta region of China. In this study, AFs were also not found in wheat [28]. Tavn et al. analyzed data on OTA contamination in grains (rice, wheat and maize) collected from six Chinese provinces, and found that the maximum OTA levels in wheat were 1.5 μg/kg [29]. This was similar to our results. In general, the result showed a very low incidence of AFs and OTA contamination in wheat samples, verifying the low risk of wheat used for soy sauce aroma-type baijiu production.

### 2.2. AFs and OTA Content in Daqu Samples

**Figure 2:**
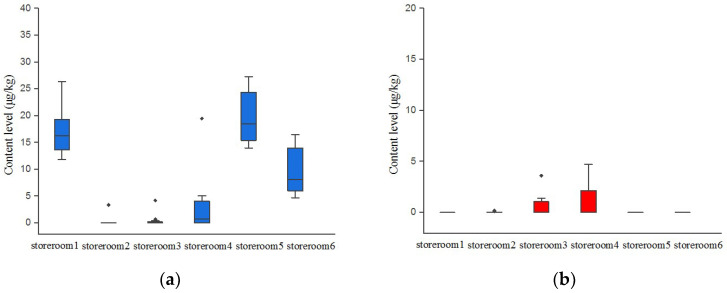

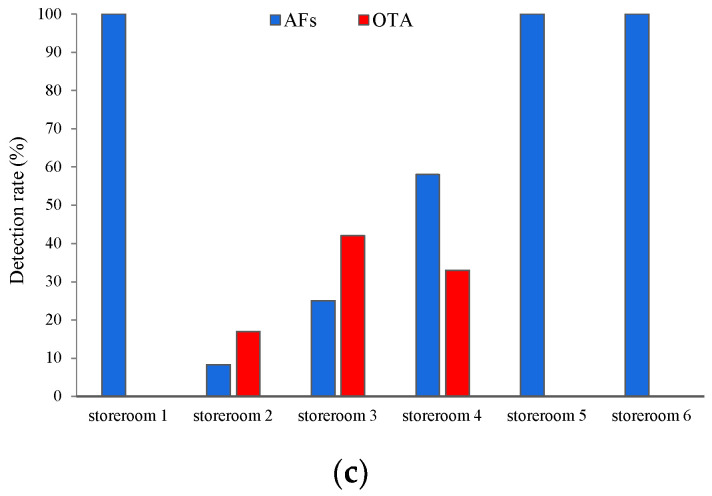
(**a**) Distribution of AFs content level in daqu samples detected by ELISA; (**b**) Distribution of OTA content level in daqu samples detected by ELISA; (**c**) detection rate (*n*%) of AFs and OTA in daqu samples.

Daqu is a traditional fermentation starter of soy sauce aroma-type baijiu made of wheat and used throughout the fermentation process of making baijiu [15,30]. Thus, it is the essential material for soy sauce aroma-type baijiu production. In this paper, daqu was also the critical monitoring point because it was fermented spontaneously under high temperatures and may be contaminated by mycotoxins [20]. We preliminarily tracked AFs and OTA concentration levels of daqu samples in six storerooms by the ELISA method (Figure 2). Among the six storerooms, storerooms 1, 4, 5 and 6 were contaminated by AFs. Storerooms 1, 5 and 6 had the highest detection rates of AFs (100%), with concentrations of 11.8–26.4 µg kg^−1^, 14.0–27.3 µg kg^−1^ and 4.7–16.5 µg kg^−1^, respectively. It was also noticed that OTA detection was only positive in storerooms 3 and 4, with an average content below 5 μg/kg that agreed with the daqu OTA values of from a previous study [20]. In general, it can be observed clearly that the positive rates and content level of AFs were higher than OTA in daqu samples, reflecting a comparatively higher potential risks of AFs contamination in daqu. Similar results were detected by Li et al., namely that the OTA content of two daqu samples was 1.4 and 1.6 μg/kg, while AFB1 was 2.5 and 2.6 μg/kg, respectively [31].

**Figure 3:**
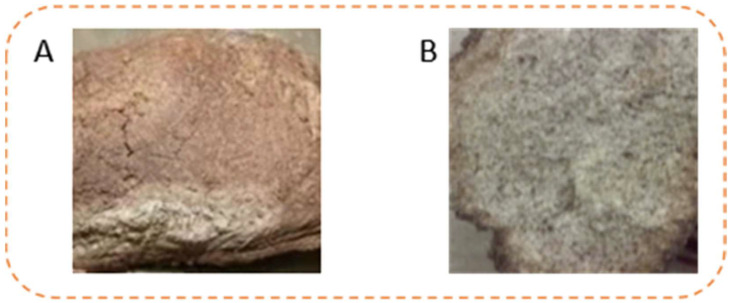
Fermented daqu (**A**) Positive daqu; (**B**) moldy, abnormal daqu.

**Figure 4:**
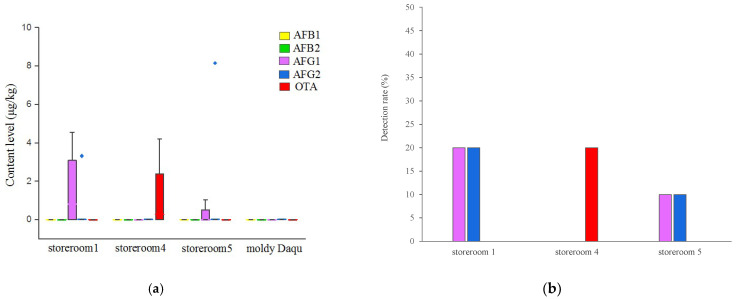
(**a**) Distribution of AFB1, AFB2, AFG1, AFG2 and OTA content level in positive daqu samples by UPLC-MS/MS; (**b**) Detection rate (*n*%) of AFG1, AFG2 and OTA and OTA in positive daqu samples.

To further confirm our findings, the AFs and OTA content in 10 positive daqu (Figure 3A) samples from storerooms 1, 4 and 5 were accurately quantified by UPLC–MS/MS (Figure 4). No AFB1 or AFB2 were found in any of the tested daqu samples. AFG1 and AFG2 was detected in 30% of the daqu samples, with the highest content being 4.56 μg/kg and 8.14 μg/kg respectively. OTA was detected in 20% of the daqu samples, with the highest content being 0.56~4.20 μg/kg, below the maximum limit of the Chinese national standard [27].

The microbial environment of moldy daqu (Figure 3A) is conducive to the variation of molds to produce mycotoxins [32]. Consequently, we inferred that moldy daqu is more easily invaded by fungi and more given to the production of mycotoxins than normal daqu. Furthermore, the content of AFs and OTA in 10 moldy daqu samples were determined by UPLC–MS/MS. The results showed that none of the AFs or OTA were detected in the 10 abnormal samples. Thus, the low risk of AFs and OTA in daqu used for soy sauce aroma-type baijiu production was further verified.

### 2.3. AFs and OTA Content in Alcoholic Fermentative Grains Samples

Alcoholic fermentative grains is one of the critical materials in the production process, of which the safety is very important. In this study, we assessed the AFs and OTA content of 10 normal alcoholic fermentative grains samples used for production, and 10 abnormal moldy alcoholic fermentative grains samples by UPLC–MS/MS method, respectively (Table 2). The results showed that AFB2, AFG1, AFG2, and OTA were rarely detected in alcoholic fermentative grains samples and AFB1 was not found—more favorable results, even, than from the previous study [33]. Pang, X. et al. reported that alcoholic fermentative grains of Qingxiangxing baijiubaijiu were positive for AFB1 at a concentration of 1.31~1.48 µg/kg. This may be due to the different production conditions and climates between soy sauce aroma-type bbaijiu and Qingxiangxing bbaijiu. Despite the difference in content level, AFs and OTA content levels in normal and abnormal moldy alcoholic fermentative grains samples were below the maximum limit standard [27]. It was concluded there was the low risk of AFS and OTA contamination in alcoholic fermentative grains.

### 2.4. AFs and OTA Content in Crude Baijiu Samples

The brewing cycle of soy sauce aroma-type baijiu includes seven different fermentation rounds, wherein each round can produce a kind of fresh liquor, also called crude baijiu. Generally, the finished baijiu is made by blending the ageing crude baijiu. Thus, the quality of finished baijiu is directly dependent on that of the crude baijiu. In other words, the occurrence of mycotoxins in crude baijiu will directly endanger consumers health. The contamination occurrence of AFs and OTA in crude baijiu were investigated by the UPLC-MS/MS method. The results showed there were no positive crude baijiu samples for AFs and OTA, indicating a very low incidence of AFs and OTA contamination brought by crude baijiu.

The results showed that there were positive of mycotoxins detected in wheat, daqu and alcoholic fermentative grains, but no mycotoxins were detected in a crude baijiu. This suggests that mycotoxins can transfer little from raw materials to crude baijiu in the distillation process. Relevant studies have shown that some mycotoxins can be metabolized to less toxic compounds or adsorbed by the spent grains in the course of the brewing process [34,35]. This coincides with the result reported by Zhu W et al. that OTA was not detected in soy sauce aroma-type baijiu [19].

### 2.5. Isolation and Screening of Fungal Communities in Daqu

Based on the analysis results above, the detection rate of AFs (AFG1, AFG2) and OTA in daqu samples were 30% and 20% respectively, while that of the wheat samples was very low. Thus, mycotoxins in wheat contribution some small amount to daqu produced from it. Daqu is rich in microbes in the fermentation process [36], and mycotoxins may originate from it. In our study, the molds of 10 positive soy sauce aroma-type daqu samples that were isolated and screened, and microbial community structure using a high-throughput sequencing (Table 3) provides a detailed identification description of the fungi species found in the positive daqu samples. The Results showed 12 genera were identified from the fungal strains isolated, in which no mycotoxins producers were found. Furthermore, the fungal communities of abnormal moldy daqu samples were isolated and screened. Forty-nine fungal genera were identified from abnormal moldy daqu samples (Table 4). Except for one Aspergillus flavum and one Penicillium flavum, the other isolates were Penicillium and Monascus. In addition, no AFs and OTA were detected in the metabolites of the isolates. Therefore, fungi presented in daqu samples, showed a very low potential to produce mycotoxins. In 2017, a total of 11 and 18 mold genera were identified from the five soy sauce aroma-type daqu samples by Qiu et al., and no data were found for potential mycotoxins-producing fungi [37]. This was also demonstrated in a report from Wang et al. [38].

## 3. Conclusions

In the fermentation of daqu and alcoholic fermentative grains, the fungal community structure can be affected by multiple factors arising from slight changes due to complications in the soy sauce aroma-type baijiu production process, which may cause mycotoxins contamination. This paper presented a food safety assessment system for the entire baijiu production process, to comprehensively analyze the content of AFs and OTA in one crucial step along the whole production process—“from raw material to liquor”—for the first time. The safety of these biological toxins was further evaluated in positive samples by microbial isolation and identification. The research results demonstrated that the risk of mycotoxins in the production process is very low, and showed the sampled soy sauce aroma-type baijiu had safe levels of these contaminants. In conclusion, a risk monitoring system was innovatively tested to ensure the quality and safety of soy sauce aroma-type baijiu in this study.

## 4. Materials and Methods

AFB1, AFB2, AFG1, AFG2, and OTA standards were purchased from Sigma-Aldrich (St. Louis, MO, USA). LC-MS grade methanol (MeOH) was obtained from ANPEL Instrument Inc. (Shanghai, China). LC-MS grade acetonitrile (ACN), formic acid (FA) were obtained from Thermo Fisher Scientific (Waltham, MA, USA). Analytical-grade ammonia, sodium chloride (NaCl), magnesium sulfate (MgSO_4_), Sodium Nitrate (NaNO_3_), ferric chloride (FeCl_3_), Ferrous sulfate (FeSO_4_), Zinc sulphate (ZnSO_4_), Cupric sulfate (CuSO_4_), Phenol, Trichloromethane were obtained from Sinopharm Inc. (Shanghai, China). The ELISA Kit of AFBs and OTA were purchased from Huaan Magnech Bio-tech Co., Ltd. (Beijing, China).

A Multi-tube vortex generator was purchased from tallboys (Thorofare, NJ, USA). The Mycotoxins detector was purchased from Femdetection Biotech Co., Ltd. (Shanghai, China). A high-speed freezing centrifuge was purchased from Hettich (MIKRO220R, Kirchlengern, Germany). Ultrasonic Cleaner was purchased from Kun Shan Ultrasonic Instruments Co., Ltd. (KQ-500DA, Kunshan, China). A Vortex mixer was purchased from Scientific Industries (Vortex-Genie2, Bohemia, NY, USA). The ProElut™PXA (60 mg/3 mL) column was purchased from Dikma Technologies Inc. (Beijing, China), and a 0.22-µm nylon filter was purchased from ANPEL Instrument Inc. (Shanghai, China). Ultra-pure water (18 MΩ cm at 23 °C) was prepared from distilled water using a Milli-Q purification system (Millipore, St. Louis, MO, USA).

Analyses were performed on a Thermo Scientific Q Exactive^TM^ mass spectrometer including an Accela pump. A heated electrospray ionization ion source was used for the ionization of target compounds. Data acquisition, peak integration, and calibration were performed using the Xcaliburw4.0 software (Thermo Scientific, Waltham, MA, USA).

### 4.1. Samples Collection

A total of 129 samples were collected from the town of Moutai, in Guizhou province, in 2017, including 69 normal daqu, 10 moldy daqu, 10 kinds of wheat, 10 normal alcoholic fermentative grains, 10 moldy alcoholic fermentative grains and 20 crude baijiu. The wheat, daqu and alcoholic fermentative grains samples were collected by experienced personnel. The wheat, daqu and alcoholic fermentative grains samples were ground to consistent and homogeneous powders (mesh < 20). The resulting samples were transferred into air-tight plastic bags, sealed and stored in dissectors −20 °C until analysis.

### 4.2. Determination of AFs and OTA in Wheat, Daqu and Alcoholic Fermentative Grains by UPLC–MS/MS

The extraction and determination of AFs and OTA was referred from the literature [20,39] the steps of which are as follows:

#### 4.2.1. Extraction of AFs

The samples, stored at −20 °C, were allowed to acclimate to room temperature. Once at room temperature, 5 g aliquots were weighed and mixed with 10 mL water and 15 mL acetonitrile in 50 mL polypropylene centrifuge tubes (Corning, Shanghai, China). The sample was vortexed for 2 min and then subjected to ultrasound in an ultrasonic generator (Kun Shan Ultrasonic Instruments Co., Ltd., Kunshan, China) for 3 min before the addition of 1 g NaCl (Sinopharm Inc., Shanghai, China). and 4 g anhydrous MgSO_4_ (Sinopharm Inc., Shanghai, China). After this, the mixture was vortexed for 1 min before centrifugation at 4000 rpm for 8 min. Then 8 mL of supernatant was transferred into a 10 mL centrifuge tube with 0.15 g N-Propylethane-1,2-diamine (Sinopharm Inc., Shanghai, China) and 0.9 g anhydrous MgSO_4_. Then, after vortexing for 1 min and centrifugation at 4000 rpm for 8 min, 5 mL of the supernatant was transferred into a 20 mL thread screw neck vial (ANPEL Instrument Inc., Shanghai, China). Finally, the liquid was dried by nitrogen gas and reconstituted with 0.5 mL of 50% methanol (ANPEL Instrument Inc., Shanghai, China) with 0.05% formic acid (Thermo Scientific, Massachusetts, USA), through a 0.22 µm nylon filter (ANPEL Instrument Inc., Shanghai, China) before analysis with UPLC–MS/MS (Thermo Scientific, Massachusetts, USA).

#### 4.2.2. Extraction of OTA

The samples stored at −20 °C were allowed to acclimate to room temperature. Once at room temperature, 3 g powder was weighed and mixed with 12 mL 75% acetonitrile (Thermo Scientific, Massachusetts, USA) with 0.25% tween80 (Sigma-Aldrich, St. Louis, MO, USA). in 50 mL polypropylene centrifuge tubes. The sample was vortexed for 1 min and then subjected to ultrasound in an ultrasonic generator for 30 min. After the ultrasonic extraction, the samples were centrifuged at 12,000 rpm for 20 min to collect the supernatant. The supernatant was adjusted to pH 7.5 with aqueous ammonia (Sinopharm Inc., Shanghai, China). for the SPE clean-up.

The SPE columns were conditioned with 5 mL of methanol followed by 5 mL of Milli-Q water. Five milliliters of supernatant was passed through these columns and washed with cleaning solution, 5 mL of Milli-Q water, and 5 mL of methanol in turn, at a flow rate of 1 drop/s, and eluted with 6 ml methanol/formic acid (96/4, *v*/*v*) solution after vacuum drying. Finally, the eluate was dried by nitrogen gas and reconstituted with 1 mL 40% acetonitrile, through a 0.22 µm nylon filter before analysis with UPLC–MS/MS.

#### 4.2.3. Analysis of AFs

The separation was performed at 35 °C using an ACQUITY UPLC^®^ BEH C18 (Waters, Milford, CT, USA) column (100 mm × 2.1 mm, 1.7 mm) at a flow rate of 0.3 mL/min. The injection volume was 1 mL. The binary mobile phases consisted of (A) methanol (B) water with the gradient elution program as follows: 0 min 95% B, 4.5 min 20% B, 4.51 min 95% B, 6 min 95% B, 13 min 95% B.

The source was operated in a positive (ESI^+^) mode at a desolvation temperature of 500 °C with N_2_ as the nebulizer and the source temperature was kept at 150 °C. The capillary voltage was maintained at 3.2 kV and the extractor voltage was set at 3.0 kV. Ultrapure nitrogen was used as desolvation gas with a flow of 40 arb (1 arb ≈ 0.3 L/min). Argon was used as the collision gas, at a flow of 40 arb.

#### 4.2.4. Analysis of OTA

Separation was performed at 35 °C using an ACQUITY UPLC^®^ BEH C18 (Waters, Milford, CT, USA) column (100 mm × 2.1 mm, 1.7 mm) at a flow rate of 0.3 mL/min. The injection volume was 5 mL. The binary mobile phases consisted of (A) water containing 0.1% (*v*/*v*) of FA and (B) ACN with the gradient elution program as follows: 0 min 5% B, 5 min 35% B, 7 min 70% B, 10 min 100% B, 14 min 100% B, 15.0 min 5% B, 18.0 min 5% B.

The source was operated in a positive (ESI^+^) mode at a desolvation temperature of 350 °C with N_2_ as the nebulizer and the source temperature was kept at 120 °C. The capillary voltage was maintained at 3.5 kV and the extractor voltage was set at 2.8 kV. Ultrapure nitrogen was used as desolvation gas with a flow of 40 arb (1 arb ≈ 0.3 L/min). Argon was used as collision gas at a flow of 40 arb.

### 4.3. Determination of AFs and OTA in Daqu by ELISA

The daqu samples stored at −20 °C were allowed to acclimate to room temperature. Once at room temperature, 4 g powder was weighed and mixed with 4.2 mL water (0.1% tween80, 1% formic acid) in 50 mL polypropylene centrifuge tubes. The mixture was vortexed for 5 min and soak for 2 h, add 11.2 mL acetonitrile (0.1% tween80, 1% formic acid), vortex for 5 min. Then it was extracted in the ultrasonic device at 40 °C for 40 min, after the ultrasonic extraction the samples were centrifuged for 10 min to collect the supernatant in 10 mL polypropylene centrifuge tubes for ELISA analysis (Huaan Magnech Bio-tech Co., Ltd., Beijing, China). The protocols specified by the manufacturers of the ELISA kits were followed.

### 4.4. Determination of AFs and OTA in Crude Baijiu Grains

Fifty milliliters of crude baijiu was taken into a round-bottomed flask and evaporated under reduced pressure at 50–60 °C. The residue was dissolved in 5 mL of acetonitrile/water (50/50, *v*/*v*), the solution was transferred into a thread screw-neck vial, and adjusted to pH 7.0 with aqueous ammonia for the SPE clean-up. The SPE clean-up process was the same as for the solid. The detection method was the same as in Section 4.2.3 and Section 4.2.4.

### 4.5. Fungi Isolated and Identified from Daqu

#### 4.5.1. Fungi Isolated from Daqu

A volume of 90 mL normal saline was added into a 250-mL triangular flask and sterilized. Then, 10.0 g daqu was weighed and put into the sterile triangular flask. This was vigorously shaken in a vortex oscillator (speed of 200 rpm) (Tallboys, New Jersey, USA) for 1 h. Next, 0.1 mL of the mixture was taken into a 2-mL sterilizing centrifuge tube (ANPEL Instrument Inc., Shanghai, China) and diluted with 0.9 mL sterile normal saline to obtain the diluent of dilution gradient of 10^−2^. Bacterial solutions, with dilution gradients of 10^−3^, 10^−4^, 10^−5^, were stepwise diluted by the above method. Then, dilutions were plated evenly on PDA culture media, to which were added ampicillin (1‰) (Sigma-Aldrich, St. Louis, MO, USA) and chloramphenicol (Sigma-Aldrich, St. Louis, MO, USA) and then they were incubated at 30 °C for 2~3 days. According to colony morphology and reproductive status, the separable fungal communities in culture media were selected, planted point by point, and lineated purification incubation on PDA culture media was conducted three times. Pure colonies identified preliminarily by colony morphology were picked out and preserved in PDA slants.

#### 4.5.2. Toxigenic Ability of Identified Strains

The 12 isolates were incubated on fresh PDA cultures for 3 days at 30 °C. Then, the spore suspensions were washed using sterile normal saline and adjusted to a cell density of 10^5^~10^6^ by counting board under a microscope and 5 μL were taken for inoculation in a YES toxigenic medium (yeast extract 20 g, sucrose 150 g, MgSO_4_·7H_2_O 0.5 g(Sinopharm Inc., Shanghai, China), ZnSO_4_·7H_2_O 0.01 g (Sinopharm Inc., Shanghai, China), CuSO_4_·5H_2_O 0.005 g (Sinopharm Inc., Shanghai, China), agar 20 g (Sinopharm Inc., Shanghai, China) and l L distilled water) and a CYA toxigenic medium (NaNO_3_ 3 g, K_2_HPO_4_ 1 g (Sinopharm Inc., Shanghai, China), KCl 0.5 g (Sinopharm Inc., Shanghai, China), MgSO_4_·7H_2_O 0.5 g, FeSO_4_·7H_2_O 0.01 g (Sinopharm Inc., Shanghai, China), ZnSO_4_·7H_2_O 0.01 g, CuSO_4_·5H_2_O 0.005 g, yeast extract (difco) 5 g, sucrose 30 g, agar 20 g and l L distilled water) and cultured at 30 °C for 7 days. The mycelium products were extracted and quantitatively detected by UPLC-MS/MS.

#### 4.5.3. Strains Identified from Daqu

For the extraction of ribosomal DNA fragments, spores on the activated PDA plate were eluted with 1.5 mL methanol and collected in a centrifugal tube to centrifugate for 2 min at 12,000 rpm. The formed supernatant was discarded after centrifugation. The precipitate was washed by adding 1 mL Milli-Q water and centrifuged for 2 min at 12,000 rpm. The bacterial precipitate was obtained after centrifugation, followed by the addition of 0.2 mL Milli-Q water, 0.3-g glass beads (diameter 0.4 mm) and 0.3 mL of a phenol(Sigma-Aldrich, St. Louis, MO, USA):chloroform(Sigma-Aldrich, St. Louis, MO, USA) (1:1) mixed solution and then broken for 30 s by a cell analyzer. After that, 0.2 mL ultrapure water was added, mixed equally, and centrifuge for 2 min at 12,000 rpm. The supernatant was obtained and twice the volume of ice ethanol was added and left to stand for 2 h at 4 °C before being centrifuged for 15 min at 12,000 rpm. The supernatant formed was discarded again after centrifugation. The precipitate was dried under vacuum and dissolved using 30~50 μL Milli-Q water.

PCR amplification was performed with an ITS sequence experiment, amplified using ITS1 and ITS4 primer. The reaction system used was as follows: ITS: 5 min at 94 °C; 94 °C 30 s, 50 °C 30 s, 72 °C 60 s, for 30 cycles; the composition of the 25 μL of reaction system used was 10.5 μL sterile water, 12.5 μL mix enzyme, 0.5 μL ITS1, 0.5 μL ITS4 and 1 μL template.

The recovery and sequencing of the PCR amplification products consisted of electrophoresis onto 1% agarose gel. Then, the target product was sequenced at the Beijing Genomics Institute.

## Figures and Tables

**Table 1:** AFs and OTA content in 10 wheat samples detected by UPLC-MS/MS.

	AFB1 (μg/kg)	AFB2 (μg/kg)	AFG1 (μg/kg)	AFG2 (μg/kg)	OTA (μg/kg)
positive *n* (%)	0/10(0)	0/10(0)	0/10(0)	0/10(0)	1/10(10)
mean ± SD (µg/kg)	nd	nd	nd	nd	1.94
range (µg/kg)	nd	nd	nd	nd	nd~1.94

**Table 2:** Accurate quantitative results of AFs and OTA in alcoholic fermentative Grains Samples.

Samples		UPLC-MS/MS (μg/kg)					
		AFB1	AFB2	AFG1	AFG2	AFs	OTA
normal fermentative grains samples	positive *n* (%)	0/10(0)	0/10(0)	1/10(10)	1/10(10)	2/10(20)	1/10(10)
	mean ± SD (µg/kg)	nd	nd	0.20 ± 0.57	0.10 ± 0.28	0.30 ± 0.61	0.19 ± 0.54
	range (µg/kg)	nd	nd	nd~1.99	nd~0.98	nd~1.99	nd~1.88
abnormal moldy alcoholic fermentative grains	positive *n* (%)	0/10(0)	1/10(10)	0/10(0)	0/10(0)	1/10(10)	1/10(10)
	mean ± SD (µg/kg)	nd	0.11 ± 0.30	nd	nd	0.11 ± 0.30	0.16 ± 0.46
	range (µg/kg)	nd	nd~1.06	nd	nd	nd~1.06	nd~1.62

**Table 3:** Fungi isolated from the positive daqu samples.

Normal Daqu	Strains Number	Species	Serial Numbers	Correlation
1	1-1	*Kluyveromyces marxianus*	CP009307.1	96%
	1-2	*Paecilomyces variotii*	FJ345354.1	99%
	1-3	*Pichia pastoris*	FR839630.1	95%
	1-4	*Paecilomyces* sp.	GQ386857.1	100%
	1-5	*Byssochlamys spectabilis*	AY373941.1	100%
	1-6	*Paecilomyces variotii*	JQ796880.1	100%
2	2-1	*Candida cellae*	GQ149495.1	94%
	2-2	*Paecilomyces variotii*	FJ345354.1	100%
	2-3	*Paecilomyces* sp.	GQ386857.1	99%
	2-4	*Byssochlamys spectabilis*	KU880725.1	100%
	2-5	*Byssochlamys spectabilis*	KC157707.1	100%
3	3-1	*Paecilomyces variotii*	JQ796880.1	99%

**Table 4:** Fungi isolated from the abnormal moldy daqu samples.

Abnormal Daqu	Strains Number	Species	Serial Numbers	Correlation
M1	M1-1	*Debaryomyces nepalensis*	KY694999.1	99%
	M1-2	*Aspergillus oryzae*	HQ285550.1	100%
	M1-3	*Aspergillus flavus*	KX067886.1	100%
	M1-5	*Penicillium aurantiovirens*	KM458838.1	100%
	M1-6	*Penicillium* sp.	LC189548.1	100%
M2	M2-2	*Penicillium* sp.	KJ527027.1	99%
	M2-3	*Penicillium* sp.	KJ527027.1	99%
	M2-4	*Hyphopichia burtonii*	KY103600.1	100%
	M2-6	*Penicillium sp. NX-deepsea*	LC189548.1	100%
M3	M3-1	*Penicillium* sp.	KR002875.1	100%
	M3-5	*Scopulariopsis brevicaulis*	KC311514.1	99%
	M3-6	*Penicillium* sp.	KJ527027.1	99%
M4	M4-2	*Hyphopichia burtonii*	KY103605.1	99%
	M4-4	*Saccharomycopsis fibuligera*	MG518197.1	100%
M5	M5-1	*Penicillium chrysogenum*	MF765611.1	100%
	M5-4	*Monascus* sp.	KY511749.1	99%
M6	M6-2	*Penicillium rubens*	LC105692.1	100%
M7	M7-4	*Monascus* sp.	KY511749.1	99%
M8	M8-1	*Penicillium rubens*	LC105692.1	99%
	M8-2	*Hyphopichia burtonii*	KY103604.1	99%
	M8-3	*Monascus fumeus*	JF776162.1	100%
M9	M9-2	*Monascus* sp.	KY511749.1	99%
M10	M10-3	*Penicillium sp. NX-deepsea*	LC189548.1	100%
	M10-4	*Paecilomyces* sp.	KF811433.1	100%
